# The Immunomodulating Effect of Baicalin on Inflammation and Insulin Resistance in High-Fat-Diet-Induced Obese Mice

**DOI:** 10.1155/2021/5531367

**Published:** 2021-05-27

**Authors:** Ji-Won Noh, Oh-Jun Kwon, Byung-Cheol Lee

**Affiliations:** Department of Clinical Korean Medicine, Graduate School, Kyung Hee University, 26 Kyungheedae-ro, Dongdaemun-gu, Seoul 02447, Republic of Korea

## Abstract

**Background:**

Obesity is a chronic low-grade systemic inflammation state, which causes insulin resistance, diabetes, and other metabolic diseases. Baicalin is known to have anti-inflammatory and antiobesity effects. In this study, we investigated the cellular and molecular immunological effects of baicalin on obesity-induced inflammation.

**Methods:**

Male C57BL/6 mice were assigned to four groups: the normal chow, high-fat diet (HFD), BC2 (HFD + baicalin 200 mg/kg), and BC4 (HFD + baicalin 400 mg/kg) group; the three groups except normal chow were fed with a high-fat diet for 8 weeks to induce obesity followed by baicalin treatment with two doses for 8 weeks. The body weight, epididymal fat weight, liver weight, food intake, oral glucose tolerance test (OGTT), oral fat tolerance test (OFTT), and serum lipids were measured. We evaluated insulin resistance by measuring the serum insulin level and homeostatic model assessment of insulin resistance (HOMA-IR). Also, the major obesity-associated immune cells including monocytes, macrophages, T lymphocytes, and dendritic cells in the blood, fat, and liver and the inflammatory and insulin signaling-related gene expressions in the fat and liver were evaluated.

**Results:**

Baicalin significantly reduced the body weight and liver weight and improved serum fasting glucose, insulin, HOMA-IR, free fatty acid, HDL cholesterol, and the levels of glucose and triglyceride at each time point in the OGTT and OFTT. In the analysis of immune cells, baicalin significantly decreased inflammatory Ly6C^hi^ monocytes, M1 adipose tissue macrophages (ATMs), and M1 Kupffer cells. On the contrary, baicalin increased anti-inflammatory M2 ATMs and liver CD4+ T cells and CD4/CD8 ratio. In the analysis of inflammatory and insulin signaling molecules, baicalin significantly downregulated the gene expression of tumor necrosis factor-*α*, F4/80, and C-C motif chemokine 2 while upregulated the insulin receptor mRNA expression.

**Conclusion:**

From these results, baicalin can be a promising treatment option for obesity and its related metabolic diseases based on its anti-inflammatory property.

## 1. Introduction

It is now generally accepted that obesity causes chronic low-grade inflammation throughout the body [1]. Local inflammation, especially in the excessive fat-deposited liver and adipose tissues, mediates systemic low-grade inflammation and results in obesity-induced insulin resistance (IR) [2, 3]. Almost immune cells including monocytes, macrophages, lymphocytes, and dendritic cells (DCs) are involved in obesity-induced inflammation, among which the number and activation level of macrophages is particularly increased [4]. In an obese state, adipose tissue macrophages (ATMs) and Kupffer cells (KCs) highly infiltrate into tissues and switch from M2 to M1 polarization [3, 5]. In addition, the imbalance between subtypes of T cells occurs, and it is involved in the recruitment and differentiation of macrophages [6, 7]. Also, the proinflammatory cytokines such as tumor necrosis factor (TNF)-*α* and C-C motif chemokine 2 (Ccl2) accelerate inflammatory response and damage insulin-signaling networks [3, 5]. Therefore, modifying the immune cell response may ameliorate obesity-induced inflammation and insulin resistance [8].

Baicalin is a bioactive flavonoid from *Scutellaria baicalensis*, which has beneficial effects on hyperglycemia and hyperlipidemia and is also linked to anti-inflammatory, antidiabetic, and antiobesity effects *in vivo* and *in vitro* [9–12]. In terms of the anti-inflammatory effects, baicalin has been reported to limit M1 polarization and promote M2 polarization *in vitro*, but most previous studies were related to inflammation caused by infection or limited to specific cytokines [13–15]. As the effects on obesity-induced sterile inflammation in a systemic manner have not been studied, it is difficult to gain the overall insight of how baicalin affects the immune system of the obese state [10, 16–18].

In this study, we examined the metabolic effects of baicalin on glucose and lipid metabolism and investigated its immunological mechanism in the blood, fat, and liver at the cellular and molecular levels.

## 2. Materials and Methods

### 2.1. Animal Models and Experimental Design

Six-week-old male C57BL/6 mice (Central Experiment Animals, Korea) weighing 19–21 g were used. Mice were kept in an animal room under the standard 12-hour light and dark cycle with 40–70% humidity and allowed *ad libitum* access to food and water. After adaptation for a week, the mice were divided into four groups of six mice per group: the normal group (NC group), HFD group, BC2 group, and BC4 group. Body weight of each mouse was recorded weekly before the morning feeding from baseline to the last sampling using an electronic scale (CAS 2.5D, Korea). Before and after the morning feeding, food was weighed to record the amount of food consumed. To compare the obese with the lean, the mice were assigned to two groups and fed the normal chow or high-fat diet containing 60% fat (HFD; Research Diets) for 8 weeks. In the following 8 weeks, the HFD-fed mice except normal chow were divided into 3 groups and additionally fed normal saline, 200 mg/kg/day or 400 mg/kg/day baicalin daily using Zonde. Baicalin was purchased from abcr GmbH (Karlsruhe, Germany). Baicalin with a purity higher than 98% was used after analysis by nuclear magnetic resonance (NMR), high-performance liquid chromatography (HPLC), and mass spectrometry (MS). All animal procedures were approved by the Kyung Hee Medical Animal Research Ethics Committee (KHMC-IACUC 19-009).

### 2.2. Oral Glucose Tolerance Test (OGTT) and Oral Fat Tolerance Test (OFTT)

Fasting blood glucose (FBG) was measured from the tail veins of mice using a strip-operated blood glucose sensor (ACCU-CHEK Performa, Australia). For the OGTT, blood glucose was measured in the same manner at 30, 60, and 120 minutes after oral administration of glucose (2 g/kg body weight). After overnight fasting, triglyceride (TG) concentration was measured from a blood sample from the tail vein and the mice were gavaged with olive oil (Sigma, USA) by 2 mL/kg body weight. For the OFTT, TG measurements were taken at 120, 240, and 360 minutes using the Accutrend Plus (Roche, USA) point-of-care device and Accutrend triglyceride strips by using the Triglyceride Colorimetric Assay Kit (Cayman, USA) [19].

### 2.3. Assessment of Insulin Resistance by Measuring Insulin Level and HOMA-IR

We extracted blood from the tail vein of mice and collected them in BD Microtainer serum separator tubes. The blood samples were centrifuged at 2,000 G for 20 minutes to obtain serum. The serum insulin level was measured by using the ultrasensitive mouse insulin ELISA kit (Crystal Chem Inc., USA). The samples and insulin standards were plated into a 96-well antibody-coated microplate by 5 *μ*L each and incubated for 2 hours at 4°C. After 5 times of washing, anti-insulin enzyme conjugate was combined into each well for 30 minutes at room temperature. After 7 times of washing, enzyme substrate solution was added and incubated for 40 minutes. Then, we added reaction stop solution to each well and waited for 10 minutes. Finally, we analyzed the microplate by using the ELISA reader at 450 nm. HOMA-IR was calculated by the following formula: fasting blood glucose (㎎/㎗) × fasting blood insulin (ng/mL) × 0.0717225161669606 [19].

### 2.4. Lipid Profile and Hepatic and Renal Function Safety Test

The mice were sacrificed under anesthesia with ether, and the blood samples were collected from the heart before the sacrifice. From the blood samples collected from the heart, nonesterified fatty acid (NEFA), TG, total cholesterol, low-density lipoprotein cholesterol (LDL-C), high-density lipoprotein cholesterol (HDL-C), phospholipid, aspartate aminotransaminase (AST), alanine aminotransaminase (ALT), and creatinine levels were estimated. After centrifuging the blood at 3,000 rpm for 20 minutes, the supernatant was stored at −40°C. The serum lipids and other biochemical parameters were analyzed by using the ELISA kit (MyBioSource, USA).

### 2.5. Isolation of Stromal Vascular Cells (SVCs) and Liver Immune Cells

The epididymal fat pads were placed in a solution of phosphate-buffered saline (PBS; Gibco, USA) and 2% bovine serum albumin (BSA; Gibco, USA) and cut into discs with a diameter of 1–2 mm size using a pair of round scissors. Tissues were mixed with collagenase (Sigma, USA) and DNase I (Roche, USA) and shaken for 20–25 minutes at 37°C. Nondigested adipose tissue was removed by filtering the mixture through a 100 *μ*m filter (BD Biosciences, USA) after mixing with 2% BSA/PBS and 5 mM EDTA. After centrifuging the sample at 1,000 rpm for 3 minutes, the supernatant was discarded and the pellet was mixed with PBS and 2% fetal bovine serum (FBS; Sigma, USA). SVCs were obtained by refiltering the mixture through a 100 *μ*m cell strainer followed by centrifugation at 200 rpm for 10 minutes to pellet the SVCs. Liver tissue was prepared after PBS (pH 7.0) perfusion by needle insertion into the portal vein. Samples without gall bladder tissue were mixed with RPMI 1640 medium containing 100 mL/L fetal calf serum (FCS), squashed in a 60 mm Petri dish, and filtered through 200G stainless mesh. After adding 8 mL Percoll (final 36.3%), 9 mL PBS, and 200 *μ*L heparin, samples were centrifuged at 2,000 rpm for 20 minutes. After pouring off the supernatant containing parenchymal cells and washing with PBS, the samples were incubated in 1X ACK lysis buffer (Lonza) at room temperature for 10 minutes to dissolve red blood cells. After centrifugation at 1,500 rpm for 5 minutes with PBS, unnecessary tissue was removed using a 100 *μ*m cell strainer. Finally, nonparenchymal cells, including immune cells, were pelletized by recentrifugation at 1,500 rpm for 5 minutes [19].

### 2.6. Fluorescence-Activated Cell Sorting (FACS) Analysis of ATMs, KCs, T Lymphocytes, DCs, and Monocytes

The cell number in each EDTA sample was adjusted to 105 after counting using a Cellometer (Nexcelom Bioscience LLC, USA). After FcBlock (BD Pharmingen, USA) was added at a ratio of 1 : 100, samples were incubated for 10 minutes. Then, samples were mixed and incubated with fluorophore-conjugated antibodies for 20 minutes in the dark. To analyze ATMs, CD45-APC Cy7 (BioLegend, USA), CD68-APC (BioLegend, USA), CD11c-phycoerythrin (CD11b-PE; BioLegend, USA), and CD206-FITC (BioLegend, USA) were used. To analyze KCs, CD45-FITC, F4/80-APC, CD11c-phycoerythrin (CD11b-PE; BioLegend, USA), and CD206-FITC (BioLegend, USA) were used. To analyze T cell population, CD45-FITC (BioLegend, USA), CD3-APC (BioLegend, USA), CD4-PerCp CY5.5 (BioLegend, USA), and CD8-phycoerythrin (BioLegend, USA) were used. Finally, to analyze monocytes, CD45-FITC, CD11b-PerCp CY5.5, and Ly6C-APC (BioLegend, USA) were used. After washing and centrifugation at 1,500 rpm, cells were analyzed using an FACSCalibur (BD Bioscience, USA) instrument and the percentages of the various cell types were determined using the FlowJo program (Tree Star, Inc., USA).

### 2.7. RNA Extraction and Real-Time PCR

The epididymal fat pads and liver were dissected from the mice. Samples were wrapped in aluminum foil, placed in liquid nitrogen, and defrosted at −70°C. They were pulverized with 300 *μ*L of ZR RNA buffer and then centrifuged at 1,000 rpm. The supernatant was transferred to Zymo-Spin III columns that were placed in 2 mL collection tubes, and two washes with 350 *μ*L of RNA wash were performed. After samples were centrifuged at 1,000 rpm with elution buffer, the eluted RNA was stored at −70°C until analysis. Gene expression was analyzed by quantitative real-time polymerase chain reaction (qRT-PCR). cDNA was synthesized from 1 mg RNA using the Advantage RT-for-PCR Kit (Clontech, USA) with 10 nM dNTPs, recombinant RNase inhibitor, MMLV reverse transcriptase, and 5x reaction buffer. After reaction at 42°C for 60 minutes and 94°C for 5 minutes, qRT-PCR was performed in a reaction mixture comprising 2x SYBR reaction buffer, primers, and distilled H_2_O using a 7900HT Fast Real-Time PCR System (Applied Biosystems^®^, Waltham, MA, USA) [19]. Sequences of the primers used for amplification are as follows: TNF-*α*, 5′-TTCTG TCTAC TGAAC TTCGG GGTGA TCGGT CC-3′ and 5′-GTATG AGATA GCAAA TCGGC TGACG GTGTG GG-3′; F4/80, 5′-CTTTGGCTATGGGCTTCCAGTC-3′ and 5′-GCAAGGAGGACAGAGTTTATCGTG-3′; Ccl2, 5′-AGGTCCCTGTCATGCTTCTGG-3′ and 5′-CTGCTGCTGGTGATCCTCTTG-3′; glyceraldehyde-3-phosphate dehydrogenase (GAPDH, housekeeping gene), 5′-AGTCCATGCCATCACTGCCACC-3′ and 5′-CCAGTGAGCTTCCCGTTCAGC-3′. The threshold cycle for each gene was converted to a relative quantitation measure based on the expression of EF-1*α* using SDS Software 2.4 (Applied Biosystems^®^, Waltham, MA, USA). Fold-change values were standardized based on the expression in the NC group as 1.

### 2.8. Statistical Analysis

Statistical analyses were performed with GraphPad PRISM 5 (GraphPad Software Inc., San Diego, USA). Groups were analyzed by one-way analysis of variance (ANOVA) and Tukey's post hoc test. Results are denoted by means ± standard error of the mean (SEM); two-tailed *p* values of <0.05 were considered significant. Significant differences from the HFD group are represented by asterisks:  ^*∗*^ for *p* < 0.05;  ^*∗∗*^ for *p* < 0.01; and  ^*∗∗∗*^ for *p* < 0.001.

## 3. Results

### 3.1. Effects of Baicalin on Body Weight, Epididymal Fat Pads, and Liver Tissue

**Figure 1:**
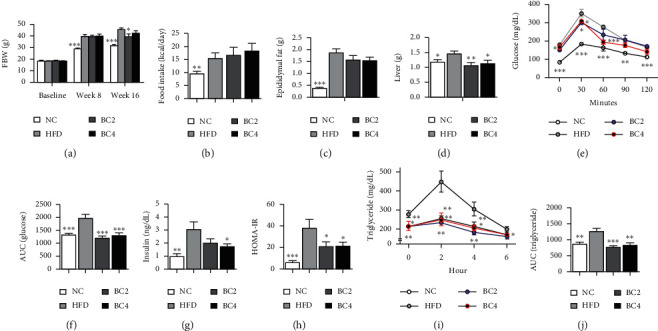
Effects of baicalin on (a) body weight, (b) food intake, (c) epididymal fat weight, (d) liver weight, (e) oral glucose tolerance test (OGTT) results, (f) area under the curve (AUC) of OGTT, (g) serum insulin level, (h) HOMA-IR, (i) oral fat tolerance test (OFTT) results, and (j) AUC of OFTT. Data shown are means ± standard errors of the mean (SEM). ^*∗*^*p* < 0.05, ^*∗∗*^*p* < 0.01, and ^*∗∗∗*^*p* < 0.001 compared with the HFD group. *n* = 6 mice per group.

Mice in the HFD group consumed more daily food intake than those in the NC group (15.42 ± 2.17 g vs. 9.49 ± 0.86 g, *p* < 0.01) and became obese by HFD for 8 weeks (Figures 1(a) and 1(b)). However, comparing the BC2 group with the HFD group, the mean body weight was significantly decreased by baicalin (39.34 ± 2.59 g vs. 45.53 ± 1.26 g, *p* < 0.05) (Figure 1(a)). The epididymal fat weights of the BC2 and BC4 groups were decreased compared with that of the HFD group (Figure 1(c)). The liver weights of the BC2 and BC4 groups were significantly decreased by baicalin compared with the HFD group (1.05 ± 0.10 g in the BC2 group vs. 1.44 ± 0.09 g in the HFD group, *p* < 0.01; 1.12 ± 0.10 g in the BC4 group vs. 1.44 ± 0.09 g in the HFD group, *p* < 0.05) (Figure 1(d)).

### 3.2. Effects of Baicalin on Glucose and Lipid Profiles

The BC2 group showed a significantly lowered FBG level compared with the HFD group (150.6 ± 8.44 mg/dL vs. 177.3 ± 9.12 mg/dL, *p* < 0.05) (Figure 1(e)). The FBC level of the BC4 group was not significantly different from that of the HFD group. To demonstrate the effects of baicalin in glucose metabolism, we performed the OGTT. Comparing BC2 and BC4 groups with the HFD group, the glucose levels after 30 min and 60 min were significantly decreased by baicalin (30 min: 301 ± 10.50 mg/dL in the BC2 group vs. 351 ± 17.34 mg/dL in the HFD group, *p* < 0.05; 309.6 ± 7.74 mg/dL in the BC4 group vs. 351 ± 17.34 mg/dL in the HFD group, *p* < 0.05; 60 min: 234.8 ± 26.96 mg/dL in the BC2 group vs. 277 ± 7.69 mg/dL in the HFD group, *p* < 0.05; 195 ± 11.60 mg/dL in the BC4 group vs. 277 ± 7.69 mg/dL in the HFD group, *p* < 0.001) (Figure 1(e)). The BC4 group had significantly decreased the area under curve (AUC) compared with the HFD group (839.0 ± 25.28 vs. 1004.5 ± 40.05, *p* < 0.001) (Figure 1(f)).

**Figure 2:**
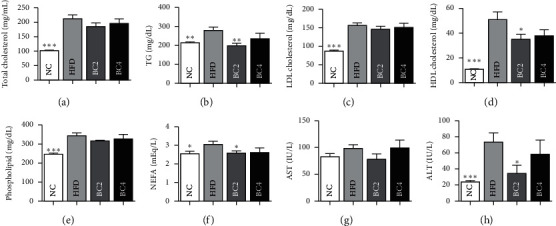
Effects of baicalin on (a) total cholesterol, (b) triglyceride, (c) LDL cholesterol, (d) HDL cholesterol, (e) phospholipid, (f) nonesterified fatty acid (NEFA), (g) aspartate aminotransferase (AST), and (h) alanine aminotransferase (ALT) levels. Data shown are means ± standard errors of the mean (SEM). ^*∗*^*p* < 0.05, ^*∗∗*^*p* < 0.01, and ^*∗∗∗*^*p* < 0.001 compared with the HFD group. *n* = 6 mice per group.

To show the effect of baicalin on lipid metabolism, we analyzed NEFA, TG, TC, LDL-C, HDL-C, and phospholipid levels, which increased due to obesity. Mice in the BC2 group had significantly lower levels of NEFA, TG, and HDL-C compared with the HFD group (NEFA: 2.59 ± 0.11 mEq/L vs. 3.05 ± 0.17 mEq/L, respectively, *p* < 0.05; TG: 35.2 ± 4.03 mg/dL vs. 50.89 ± 6.31 mg/dL, respectively, *p* < 0.05; HDL-C: 198.6 ± 12.56 mg/dL vs. 279.2 ± 16.96 mg/dL, respectively, *p* < 0.01) (Figures 2(b), 2(d), and 2(f)). BC2 also lowered the other serum lipid levels, but it was not significant. Mice in the BC4 group had lower serum lipid levels compared with the HFD group (Figures 2(a), 2(c), and 2(e)). In the OFTT, the HFD group had higher TG levels than the NC group at all time points except for the 6 hr time point. Mice in the BC2 group showed significantly decreased TG levels at all time points compared with the HFD group (0 hr, *p* < 0.01; 2 hr, *p* < 0.01; 4 hr, *p* < 0.01; 6 hr, *p* < 0.05) (Figure 1(i)), which were even lower than those in the NC group. BC4 also significantly lowered TG levels at 0, 2, and 4 hr compared with the HFD group (0 hr, *p* < 0.05; 2 hr, *p* < 0.01; 4 hr, *p* < 0.05) (Figure 1(g)). Mice in the HFD group had higher AUCs compared with the NC group. The AUCs of both BC2 and BC4 groups were significantly reduced compared with that of the HFD group (1216.8 ± 66.5 and 1306.4 ± 105.1 vs. 1982.2 ± 130.1, respectively, *p* < 0.001) (Figure 1(j)).

### 3.3. Effects of Baicalin on Serum Insulin Level and HOMA-IR

To evaluate the effects on insulin resistance, we measured the fasting insulin level and HOMA-IR. The insulin level was remarkably higher in the HFD group compared with the NC group (3.03 ± 0.62 ng/mL vs. 0.98 ± 0.20 ng/mL, *p* < 0.01). We observed decrease in both BC2 and BC4 groups, but only the BC4 group showed significant difference compared with the HFD group (1.76 ± 0.20 ng/mL vs. 3.03 ± 0.62 ng/mL, *p* < 0.05) (Figure 1(g)). Similar pattern was observed in HOMA-IR. HOMA-IR of the HFD group was significantly higher than that of the NC group (38.28 ± 7.78 vs. 6.15 ± 1.60, *p* < 0.001). Both BC2 and BC4 groups showed a significant decrease in HOMA-IR compared with the HFD group (21.02 ± 4.45 and 21.71 ± 3.05 vs. 38.28 ± 7.78, *p* < 0.05) (Figure 1(h)).

### 3.4. Effects of Baicalin on Hepatic and Renal Function

For examining the safety of baicalin treatment, serum AST and ALT levels were estimated after 16 weeks. Mice in the HFD group had a higher level of ALT compared with the NC group. BC2 lowered the ALT level as half significantly compared with the HFD group (34.4 ± 10.25 U/L vs. 73.78 ± 11.11 U/L, *p* < 0.05), but it was not dose-dependent (Figure 2(h)). The levels of AST and creatinine were not different between all groups (Figure 2(g)).

### 3.5. Effects of Baicalin on Monocytes

**Figure 3:**
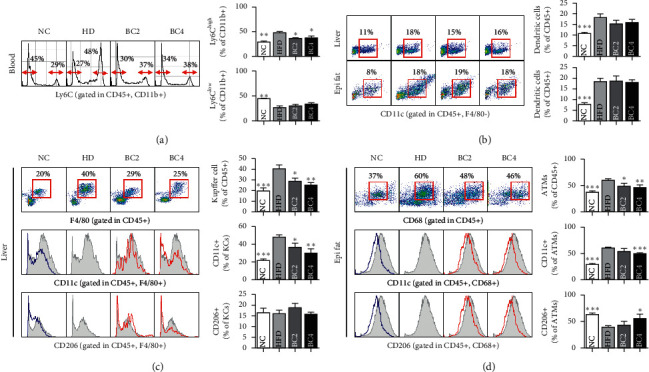
Effects of baicalin on (a) Ly6C monocytes in the CD11b+ population, (b) dendritic cells in liver and epididymal fat tissues, (c) liver Kupffer cells, CD11c+ Kupffer cells, and CD206+ Kupffer cells, and (d) adipose tissue macrophages, CD11c+ adipose tissue macrophages, and CD206+ adipose tissue macrophages. Data shown are means ± standard errors of the mean (SEM). ^*∗*^*p* < 0.05, ^*∗∗*^*p* < 0.01, and ^*∗∗∗*^*p* < 0.001 compared with the HFD group. *n* = 6 mice per group.

Mice in the HFD group had higher Ly6C^hi^ monocyte and lower Ly6C^low^ monocyte fraction compared with the NC group. BC2 and BC4 significantly lowered Ly6C^hi^ monocyte fraction compared with the HFD group (36.63 ± 1.21% and 37.68 ± 3.73% vs. 48.02 ± 2.51%, respectively, *p* < 0.05) (Figure 3(a)). BC2 and BC4 lowered Ly6C^low^ monocyte fraction compared with the HFD group without statistical significance.

### 3.6. Effects of Baicalin on ATMs and KCs

Mice in the HFD group had a significantly higher percentage of ATMs compared with the NC group. BC2 and BC4 significantly lowered the percentage of ATMs compared with the HFD group (48.38 ± 6.01% in BC2 group vs. 60.24 ± 2.76% in the HFD group, *p* < 0.05; 46.44 ± 4.89% in BC4 group vs. 60.24 ± 2.76% in the HFD group, *p* < 0.01) (Figure 3(d)). Mice in the HFD group had more CD11c+ ATMs and fewer CD206+ ATMs compared with the NC group (*p* < 0.001). However, BC4 decreased CD11c+ ATMs and increased CD206+ ATMs significantly compared with the HFD group (50.16 ± 1.07% vs. 60.24 ± 1.70%, *p* < 0.001; 55.99 ± 7.97% vs. 39.12 ± 3.04%, *p* < 0.05, respectively) (Figure 3(d)). In the liver tissue, the mice in the HFD group had higher percentages of KCs compared with the NC group. However, BC2 and BC4 lowered the percentage of KCs significantly compared with the HFD group (28.55 ± 3.15% in the BC2 group vs. 40.37 ± 3.61% in the HFD group, *p* < 0.05; 25.17 ± 2.26% in the BC4 group vs. 40.37 ± 3.61% in the HFD group, *p* < 0.01) (Figure 3(c)). Mice in the HFD group had a higher percentage of CD11c+ KCs and a lower percentage of CD206+ KCs compared with the NC group. However, BC2 and BC4 lowered the percentage of CD11c+ KCs compared with the HFD group significantly in a dose-dependent manner (36.26 ± 4.90% in the BC2 group vs. 47.72 ± 2.70% in the HFD group, *p* < 0.05; 29.95 ± 4.92% in the BC4 group vs. 47.72 ± 2.70% in the HFD group, *p* < 0.01) (Figure 3(c)). The percentage of CD206+ KCs was not significantly different among the HFD, BC2, and BC4 groups.

### 3.7. Effects of Baicalin on T Cells and DCs in Blood, Adipose Tissue, and Liver

**Figure 4:**
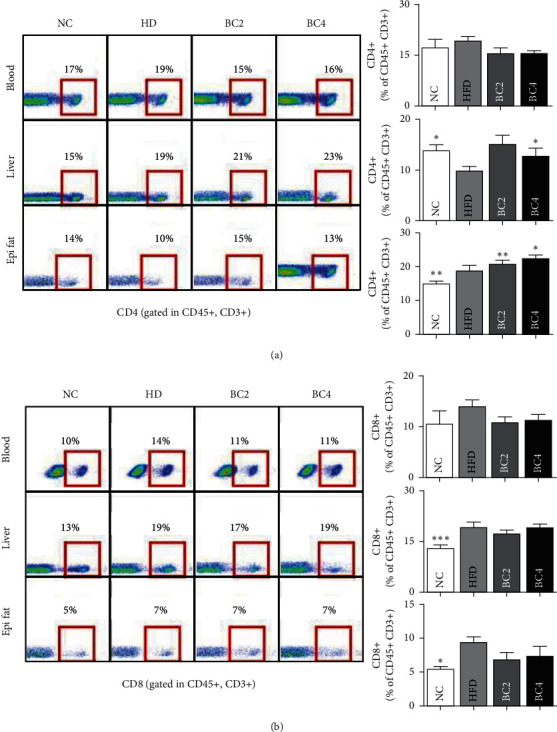
Effects of baicalin on (a) CD4+ T cells in the blood, liver, and epididymal fat pads and (b) CD8+ T cells in the blood, liver, and epididymal fat pads. Data shown are means ± standard errors of the mean (SEM). ^*∗*^*p* < 0.05, ^*∗∗*^*p* < 0.01, and ^*∗∗∗*^*p* < 0.001 compared with the HFD group. *n* = 6 mice per group.

Mice in the HFD group had a significantly higher proportion of CD4+ T cells, lower proportion of CD8+ T cells, and lower CD4/CD8 ratio compared with the NC group. In adipose tissue, BC2 and BC4 enlarged the proportion of CD4+ T cells significantly compared with the HFD group (15.23 ± 1.64% in the BC2 group vs. 9.84 ± 0.82% in the HFD group, *p* < 0.01; 12.86 ± 1.52% in the BC4 group vs. 9.84 ± 0.82% in the HFD group, *p* < 0.05) (Figure 4(a)). Regarding CD8+ T cells, the proportions in both BC2 and BC4 groups were not significantly different from those of the HFD group (Figure 4(b)). The CD4/CD8 ratio of BC2 and BC4 groups were significantly higher than that of the HFD group (2.36 ± 0.36 in the BC2 group vs. 1.38 ± 0.15% in the HFD group, *p* < 0.01; 1.90 ± 0.27 in the BC4 group vs. 1.38 ± 0.15% in the HFD group, *p* < 0.05).

In the liver, BC4 enlarged the percentage of CD4+ T cells significantly compared with the HFD group (22.64 ± 0.85% vs. 18.73 ± 1.50%, *p* < 0.05) (Figure 4(a)). Regarding CD8+ T cells, there was no change by baicalin treatment (Figure 4(b)). The CD4/CD8 ratio of BC2 and BC4 groups were significantly increased compared with that of the HFD group (1.20 ± 0.02 in the BC2 group vs. 0.97 ± 0.03 in the HFD group, *p* < 0.001; 1.19 ± 0.06 in the BC4 group vs. 0.97 ± 0.03 in the HFD group, *p* < 0.001). In blood, the populations of CD4+ and CD8+ T cells were not significantly different among all groups (Figures 4(a) and 4(b)). The number of CD45^+^ F4/80^−^ CD11c^+^ DCs in adipose and liver tissues was significantly increased by HFD, but there was no change by baicalin treatment (Figure 3(b)).

### 3.8. Effects of Baicalin on the Expression of Inflammatory and Insulin Signaling-Related Genes

**Figure 5:**
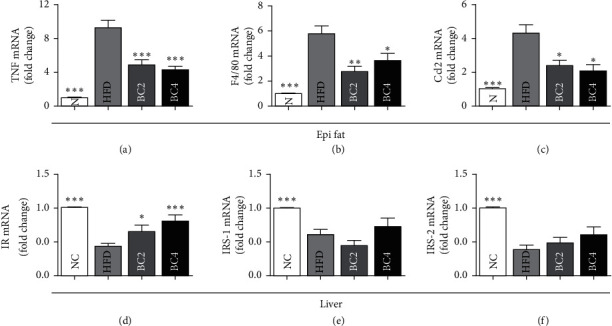
Effects of baicalin on the gene expression of (a) TNF-*α*, (b) F4/80, and (c) Ccl2 in epididymal fat pads and (d) IR, (e) IRS-1, and (f) IRS-2 in the liver. Data shown are means ± standard errors of the mean (SEM). ^*∗*^*p* < 0.05 and ^*∗∗∗*^*p* < 0.001 compared with the HFD group. *n* = 6 mice per group.

In the adipose tissue, the transcript levels of TNF-*α*, F4/80, and Ccl2 were analyzed. Mice in the HFD group showed higher levels of TNF-*α*, F4/80, and Ccl2 mRNA compared with the NC group. However, BC2 and BC4 lowered TNF-*α* mRNA levels compared with the HFD group significantly (4.84 ± 0.66 in the BC2 group vs. 9.24 ± 1.31 in the HFD group, *p* < 0.001; 4.33 ± 0.38 in the BC4 group vs. 9.24 ± 1.31 in the HFD group, *p* < 0.001) (Figure 5(a)). Both BC2 and BC4 groups had significantly lower F4/80 and Ccl2 mRNA levels than the HFD group (F4/80: 2.78 ± 0.45 in the BC2 group vs. 5.81 ± 0.93 in the HFD group, *p* < 0.001; 3.67 ± 0.61 in the BC4 group vs. 5.81 ± 0.93 in the HFD group, *p* < 0.05; Ccl2: 2.40 ± 0.34 in the BC2 group vs. 4.29 ± 0.76 in the HFD group, *p* < 0.05; 2.08 ± 0.41 in the BC4 group vs. 4.29 ± 0.76 in the HFD group, *p* < 0.05) (Figures 5(b) and 5(c)).

In the liver tissue, transcript levels of insulin receptor (IR), insulin receptor substrate-1 (IRS-1), and IRS2 were analyzed to investigate the effects of baicalin on hepatic insulin signaling pathway. Mice in the HFD group had significantly decreased IR and IRS-2 mRNA levels and increased IRS-1 mRNA level compared with the NC group (*p* < 0.001). BC2 and BC4 groups had significantly higher IR mRNA levels than the HFD group (0.65 ± 0.11 in the BC2 group vs. 0.44 ± 0.06 in the HFD group, *p* < 0.05; 0.81 ± 0.10 in the BC4 group vs. 0.44 ± 0.06 in the HFD group, *p* < 0.01) (Figure 5(d)). Transcript levels of IRS-1 and IRS-2 in BC2 and BC4 groups were not significantly different from those in the HFD group (Figures 5(e) and 5(f)).

## 4. Discussion

This is the first study to investigate the metabolic effects and immunological mechanism of baicalin on obesity-induced inflammation in cellular and genetic levels. We discovered the cellular mechanisms by modulating Ly6C^hi^ monocytes, M1 ATMs, M1 KCs, and CD4/CD8 T cells ratio and the genetic mechanism by decreasing mRNA expressions of inflammatory genes and increasing insulin signaling-related genes.

Obesity increases the risk of metabolic diseases including dyslipidemia, T2DM, nonalcoholic fatty liver disease (NAFLD), cardiovascular disease, and cancer by causing a chronic state of low-grade systemic inflammation [2, 3, 20]. Local inflammation in adipose tissue and the liver is the main contributor to systemic inflammation. The primary characteristic of obesity-induced inflammation is the extensive infiltration of macrophages into adipose tissue. Proinflammatory cytokines from ATMs damage insulin-signaling networks, aggravate infiltration, and induce proinflammatory differentiation of macrophages [3, 5]. At the same time, lipolysis in adipose tissue increases free fatty acid flux and fat oxidation in the liver, resulting in poor glucose oxidation and fatty liver. As gluconeogenesis increases, obesity-induced IR prior to beta-cell dysfunction plays a major role in the initial stage of T2DM [1, 20]. Therefore, anti-inflammatory modulation in metabolic organs is a promising treatment target of obesity and its associated metabolic diseases. In this study, we assessed the metabolic features, the major immune cells especially macrophages, and inflammatory and insulin-signaling genes of obese mice to gain insights into antiobesity and antidiabetic effects of baicalin.

Baicalin treatment significantly decreased body weight without any change in the weight of epididymal fat pads. This indicated that the anti-inflammatory properties of baicalin as reflected by improvements in glucose and lipid metabolism were not due to a loss in adipocyte volume but rather to changes in the immune cell population in the blood and adipose and liver tissues. It represented that baicalin treatment is a systemic immunomodulating treatment for obesity-associated metabolic diseases, especially T2DM and NAFLD. Mice treated with baicalin exhibited significantly lower glucose levels at 0, 30, and 60 min in OGTTs. Given that the first-phase insulin response is the principal predictor of T2DM development, these results suggest that baicalin improves glucose metabolism [21]. We also noticed that baicalin ameliorated insulin resistance by observing the decrease in the insulin level and HOMA-IR. NEFA release due to lipolysis of adipocytes inhibits insulin-mediated glucose uptake [22, 23]. Baicalin was observed to significantly reduce TG levels at all time points studied in OFTTs and the levels of NEFA and HDL-C. The observed changes in NEFA and TG but not TC are consistent with the findings of Xu et al. [24] but differ from those of Xi and colleagues. Xi et al. [17] reported that administration of baicalin for 14 weeks to obese mice increased HDL-C and reduced TC and LDL-C. Considering that hepatic lipoprotein metabolism proceeds in the order of NEFA uptake, TG synthesis, and VLDL-C hydrolysis, the results after 8 weeks are expected to be the same as Xi et al.'s result after 14 weeks. This study has a potential limitation that we had not established the positive control group. We aimed to assess the effects of baicalin on various metabolic and immunological markers for the first time and observed its beneficial actions. Further studies are required comparing baicalin with conventional medicine such as metformin or statins to obtain more robust evidence on the treatment effect of baicalin on obesity and its associated diseases.

In this study, we demonstrated that baicalin modulated the proportion of ATMs and KCs based on a decrease in Ly6C^hi^ monocytes. Ly6C^hi^ monocytes respond to lipopolysaccharides and worsen inflammation, while Ly6C^low^ monocytes remodel the inflammation sites. Ly6C^hi^ and Ly6C^low^ monocytes are more likely to differentiate into M1 and M2 macrophages, respectively, when infiltrating into local tissues. Classically activated CD11c+ macrophages (M1) produce proinflammatory cytokines such as TNF-*α* as well as a high quantity of reactive oxygen species (ROS), while alternatively activated CD206+ macrophages (M2) produce anti-inflammatory cytokines such as IL-4 and IL-10. KCs are hepatic macrophages, and the expansion of M1 KCs results in hepatic inflammation, leading independently to nonalcoholic steatohepatitis (NASH) and hepatic IR [5]. In the obese state, HFD-induced hypercholesterolemia resulted in selective expansion of Ly6C^hi^ monocytes and inhibited the conversion from Ly6C^hi^ to Ly6C^low^ monocytes [25, 26], and a shift to M1 ATMs strongly mediated the development of IR [8, 27]. However, baicalin significantly modulated the monocyte phenotype with fewer Ly6C^hi^ monocytes and more Ly6C^low^ monocytes. This change from Ly6C^hi^ to Ly6C^low^ monocytes in the blood contributed to an M2-dominant shift in ATMs and KCs. Also, baicalin significantly restricted M1 KC activation with no change in M2 KCs, suppressing the hepatic inflammation. Taken together, we discovered that baicalin modulated macrophage differentiation along with Ly6C^hi^ monocytes' decrement, leading to anti-inflammation in the adipose tissue and liver.

The distribution of T cells also affects obesity-induced inflammation and hepatic steatosis. CD4+ regulatory T (Treg) cells are known to suppress inflammation by checking ATMs, while CD8+ T cells affect M1 polarization by secreting IFN-*γ* [1, 6]. As CD4+ T cells have 4 subtypes, CD4+ Th1 cells and an imbalance between T17 and Treg cells stimulate M1 expansion. Furthermore, CD4+ Th2 cells induce M2 polarization. In this study, baicalin significantly enhanced only the CD4+ T cell population in adipose tissue with no change in the CD8+ T cell population. However, in the liver, baicalin increased the number of CD4+ T cells and inhibited CD8+ T cells significantly. Further study is needed to determine how baicalin affects subtypes of CD4+ T cells including Th1, Th2, Treg, and Th17 cells [28, 29]. DCs differentiated from monocytes are potent antigen-presenting cells that activate CD4+ T cells, especially Th1 cells, leading to M1 ATM expansion in adipose tissue. Hepatic DCs are also known to play key roles in the pathogenesis and resolution of NASH [30, 31]. In this study, baicalin did not affect the number of adipose DCs but decreased hepatic DCs slightly. This indicates that the anti-inflammatory effects are primarily due to its effects on macrophages. However, natural killer cells such as IFN-*γ*-secreting immune cells that can affect M1 macrophages should also be investigated in future studies.

Inflammatory proteins, especially Ccl2 (encoding MCP-1), recruit macrophages into obese adipose tissue and TNF-*α* further activates the inflammatory cascade, maintaining obesity-induced systemic inflammation [32]. Consistent with previous results, we demonstrated that baicalin significantly decreased the mRNA expression of Ccl2 and TNF-*α*. F4/80 is a specific marker to murine macrophages. We observed a significant decrease in F4/80 expression but no change in adipose DCs after baicalin treatment, indicating that the effects of baicalin are restricted to the ATM population, not the adipose DC population, and are mediated by the downregulation of Ccl2 and TNF-*α*. Insulin binds to the insulin receptor and activates the tyrosine kinases IRS1 and IRS2. In obesity, IR is demonstrated by the downregulation of IRS2 and persistent expression of IRS1, and inflammatory cytokines including TNF-*α* downregulate IRS2 mRNA expression [33, 34]. In this study, we observed a lower expression of IRS1 mRNA in the HFD group than that of the NC group, but no significant change in IRS1 mRNA levels after baicalin treatment. However, baicalin significantly upregulated IR mRNA expression.

Our findings suggest that oral administration of baicalin to obese mice has favorable effects on the systemic inflammatory state by reducing Ly6C^hi^ monocytes in the blood, proinflammatory macrophages, and T cells in the liver and adipose tissues, leading to improvement of obesity-induced IR, diabetes, dyslipidemia, and metabolic diseases.

## 5. Conclusion

This new finding suggests that baicalin is a promising treatment for obesity-related metabolic diseases by improving systemic inflammatory conditions, and further study including clinical trial should be required to confirm these results.

## Data Availability

The datasets used and analyzed in this study are available from the corresponding author upon reasonable request.

## References

[1] Harford K. A., Reynolds C. M., McGillicuddy F. C., Roche H. M. (2011). Fats, inflammation and insulin resistance: insights to the role of macrophage and T-cell accumulation in adipose tissue. *Proceedings of the Nutrition Society*.

[2] Conroy M. J., Dunne M. R., Donohoe C. L., Reynolds J. V. (2016). Obesity-associated cancer: an immunological perspective. *Proceedings of the Nutrition Society*.

[3] Heymsfield S. B., Wadden T. A. (2017). Mechanisms, pathophysiology, and management of obesity. *New England Journal of Medicine*.

[4] Ambreen Asghar N. S. (2017). Role of immune cells in obesity induced low grade inflammation and insulin resistance. *Cellular Immunology*.

[5] Odegaard J. I., Ricardo-Gonzalez R. R., Red Eagle A. (2008). Alternative M2 activation of Kupffer cells by PPARdelta ameliorates obesity-induced insulin resistance. *Cell Metabolism*.

[6] Nishimura S., Manabe I., Nagasaki M. (2008). CD8+ effector T cells contribute to macrophage recruitment and adipose tissue inflammation in obesity. *Nature Medicine*.

[7] McLaughlin T., Liu L. F., Lamendola C. (2014). T-cell profile in adipose tissue is associated with insulin resistance and systemic inflammation in humans. *Arteriosclerosis, Thrombosis, and Vascular Biology*.

[8] Lumeng C. N., Bodzin J. L., Saltiel A. R. (2007). Obesity induces a phenotypic switch in adipose tissue macrophage polarization. *Journal of Clinical Investigation*.

[9] Zhang J., Zhang H., Deng X. (2018). Baicalin attenuates non-alcoholic steatohepatitis by suppressing key regulators of lipid metabolism, inflammation and fibrosis in mice. *Life Science*.

[10] Li H. T., Wu X. D., Davey A. K., Wang J. (2011). Antihyperglycemic effects of baicalin on streptozotocin - nicotinamide induced diabetic rats. *Phytotherapy Research*.

[11] Lee H., Kang R., Hahn Y. (2009). Antiobesity effect of baicalin involves the modulations of proadipogenic and antiadipogenic regulators of the adipogenesis pathway. *Phytotherapy Research*.

[12] Fang P., Yu M., Zhang L. (2017). Baicalin against obesity and insulin resistance through activation of AKT/AS160/GLUT4 pathway. *Molecular and Cellular Endocrinology*.

[13] Lai Y. S., Putra R., Aui S. P., Chang K. T. (2018). M2_C_ polarization by baicalin enhances efferocytosis via upregulation of MERTK receptor. *American Journal of Chinese Medicine*.

[14] Zhu W., Jin Z., Yu J. (2016). Baicalin ameliorates experimental inflammatory bowel disease through polarization of macrophages to an M2 phenotype. *International Immunopharmacology*.

[15] Shi-Xue Dai Y. Z., Feng Y.-L., Liu H.-B, Zheng X.-B. (2013). Baicalin down-regulates the expression of macrophage migration inhibitory facotr (MIF) effectively for rats with ulcerative colitis. *Phytotherapy Research*.

[16] Kim M. E., Kim H. K., Park H. Y., Kim D. H., Chung H. Y., Lee J. S. (2013). Baicalin from Scutellaria baicalensis impairs Th1 polarization through inhibition of dendritic cell maturation. *Journal of Pharmacological Sciences*.

[17] Xi Y., Wu M., Li H. (2015). Baicalin attenuates high fat diet-induced obesity and liver dysfunction: dose-response and potential role of CaMKKbeta/AMPK/ACC pathway. *Cellular Physiology and Biochemistry*.

[18] Liao P., Liu L., Wang B., Li W., Fang X., Guan S. (2014). Baicalin and geniposide attenuate atherosclerosis involving lipids regulation and immunoregulation in ApoE-/- mice. *European Journal of Pharmacology*.

[19] Hyun-Young Na B.-C. L. (2019). Scutellaria baicalensis alleviates inuslin resistance in diet-induced obese mice by modulating inflammation. *International Journal of Molecular Sciences*.

[20] Deng T., Lyon C. J., Bergin S., caligiuri M. A., Hsueh W. A. (2016). Obesity, inflammation, and cancer. *Annual Review of Pathology: Mechanisms of Disease*.

[21] Caumo A., Luzi L. (2004). First-phase insulin secretion: does it exist in real life? Considerations on shape and function. *American Journal of Physiology-Endocrinology and Metabolism*.

[22] Paolisso G., Howard B. V. (1998). Role of non-esterified fatty acids in the pathogenesis of type 2 diabetes mellitus. *Diabetics Medicine*.

[23] Acton Q. A. (2013). *Obesity and Diabetes: New Insights for the Healthcare Professional: 2013 Edition*.

[24] Xu J., Li Y., Lou M. (2018). Baicalin regulates SirT1/STAT3 pathway and restrains excessive hepatic glucose production. *Pharmacological Research*.

[25] Swirski F. K., Libby P., Aikawa E. (2007). Ly-6Chi monocytes dominate hypercholesterolemia-associated monocytosis and give rise to macrophages in atheromata. *Journal of Clinical Investigation*.

[26] Yang J., Zhang L., Yu C., Yang X. F., Wang H. (2014). Monocyte and macrophage differentiation: circulation inflammatory monocyte as biomarker for inflammatory diseases. *Biomarker Research*.

[27] Zhang C., Wang K., Yang L. (2018). Lipid metabolism in inflammation-related diseases. *Analyst*.

[28] Kang K., Reilly S. M., Karabacak V. (2008). Adipocyte-derived Th2 cytokines and myeloid PPARdelta regulate macrophage polarization and insulin sensitivity. *Cell Metabolism*.

[29] Elisa Fabbrini M. C., Mccartney S. A., Fuchs A. (2013). Association between specific adipose tissue CD4DT-cell populationsand insulin resistance in obese individuals. *Gastroenterology*.

[30] Park M., Woo S. Y. (2016). Inflmmation in obesity. *Journal of Bacteriology and Virology*.

[31] Stefanovic-Racic M., Yang X., Turner M. S. (2012). Dendritic cells promote macrophage infiltration and comprise a substantial proportion of obesity-associated increases in CD11c+ cells in adipose tissue and liver. *Diabetes*.

[32] Rasheed Ahmad A. A.-R., Kochumon S., Akther N. (2018). The synergy between palmitate and TNF-a for CCL2 production is dependent on the TRIF/IRF3 pathway: implications for metabolic inflammation. *The Journal of Immunology*.

[33] Midori Honma S. S., Ueno Y., Murakami K. (2018). Selective insulin resistance with differential expressions of IRS-1 and IRS-2 in human NAFLD livers. *International Journal of Obesity*.

[34] Kubota T., Kubota N., Kadowaki T. (2017). Imbalanced insulin actions in obesity and type 2 diabetes: key mouse models of insulin signaling pathway. *Cell Metabolism*.

